# Prognosis prediction of icotinib as targeted therapy for advanced EGFR-positive non–small cell lung cancer patients

**DOI:** 10.1007/s10637-023-01329-8

**Published:** 2023-05-04

**Authors:** Xueyun Tan, Sufei Wang, Hui Xia, Hebing Chen, Juanjuan Xu, Daquan Meng, Zhihui Wang, Yan Li, Lian Yang, Yang Jin

**Affiliations:** 1 Department of Respiratory and Critical Care Medicine, Hubei Province Clinical Research Center for Major Respiratory Disease, NHC Key Laboratory of Pulmonary Diseases, Union Hospital, Tongji Medical College, Huazhong University of Science and Technology, 430022 Wuhan, Hubei China; 2 Hubei Province Engineering Research Center for Tumor-Targeted Biochemotherapy, MOE Key Laboratory of Biological Targeted Therapy, Tongji Medical College, Union Hospital, Huazhong University of Science and Technology, Wuhan, China; 3 Department of Radiology, Union Hospital, Tongji Medical College, Huazhong University of Science and Technology, 430022 Wuhan, Hubei China; 4 Department of Scientific Research, Union Hospital, Tongji Medical College, Huazhong University of Science and Technology, Wuhan, Hubei China; 5 Department of Pathology, Union Hospital, Tongji Medical College, Huazhong University of Science and Technology, Wuhan, China

**Keywords:** Icotinib, NSCLC, EGFR, Prognostic prediction

## Abstract

Clinical trials on icotinib, a first-generation epidermal growth factor receptor tyrosine kinase inhibitor (EGFR-TKI), have shown promising results as targeted therapy for non-small cell lung cancer (NSCLC). This study aimed to establish an effective scoring system to predict the one-year progression-free survival (PFS) of advanced NSCLC patients with EGFR mutations treated with icotinib as targeted therapy. A total of 208 consecutive patients with advanced EGFR-positive NSCLC treated with icotinib were enrolled in this study. Baseline characteristics were collected within 30 days before icotinib treatment. PFS was taken as the primary endpoint and the response rate as the secondary endpoint. Least absolute shrinkage and selection operator (LASSO) regression analysis and Cox proportional hazards regression analysis were used to select the optimal predictors. We evaluated the scoring system using a five-fold cross-validation. PFS events occurred in 175 patients, with a median PFS of 9.9 months (interquartile range, 6.8-14.5). The objective response rate (ORR) was 36.1%, and the disease control rate (DCR) was 67.3%. The final ABC-Score consisted of three predictors: age, bone metastases and carbohydrate antigen 19-9 (CA19-9). Upon comparison of all three factors, the combined ABC-score (area under the curve (AUC)= 0.660) showed a better predictive accuracy than age (AUC = 0.573), bone metastases (AUC = 0.615), and CA19-9 (AUC = 0.608) individually. A five-fold cross-validation showed good discrimination with AUC = 0.623. The ABC-score developed in this study was significantly effective as a prognostic tool for icotinib in advanced NSCLC patients with EGFR mutations.

**Supplementary Information:**

The online version contains supplementary material available at 10.1007/s10637-023-01329-8.

## Introduction

Treatment with tyrosine kinase inhibitors (TKIs) is strongly recommended for patients with advanced non–small cell lung cancer (NSCLC) harbouring epidermal growth factor receptor (EGFR) mutations that are sensitive to TKIs, such as exon 19 deletion (19-Del) and exon 21 L858R (21-L858R) [1, 2]. It has been shown that EGFR-TKIs can significantly improve the clinical outcomes, including progression-free survival (PFS), disease-free survival (DFS) and overall survival (OS), of EGFR-positive NSCLC patients [3–5]. Additionally, compared with standard chemotherapy, EGFR-TKIs displayed higher safety, better tolerability, and patients had improved quality of life when used as the first-line treatment for patients with advanced EGFR-positive NSCLC in previous studies [6–8]. Currently, third-generation EGFR-TKIs are in active clinical development, focused on controlling acquired resistance to the targeted therapy. In the past decade, a significant number of patients who followed the sequential treatment approach received first-generation EGFR-TKIs as their initial therapy [9, 10].

Icotinib is an orally ingested first-generation EGFR-TKI with potent antitumour activity and high selectivity [11, 12]. It has proven to be more effective than chemotherapy as the first-line treatment for advanced NSCLC patients with EGFR mutations in a phase III clinical trial [13]. Moreover, icotinib exceeds gefitinib as a second-line or third-line treatment for pretreated patients with advanced NSCLC [14]. Furthermore, it has been widely used in China and there is sufficient evidence of its favorable safety and tolerability profile [15, 16]. Considering the promising results and efficacy of icotinib, this study aimed to investigate an effective prognostic scoring system to predict the one-year PFS for advanced NSCLC patients with EGFR mutations treated with icotinib as an EGFR-TKI targeted therapy.

Serum tumor markers (STMs) and other combined laboratory indexes have been widely used clinically as diagnostic biomarkers and to determine prognosis of cancer patients. In our study, carcinoembryonic antigen (CEA), carbohydrate antigen 125 (CA-125), and carbohydrate antigen 19 − 9 (CA19-9) were included due to their marked importance in lung cancer. However, STMs have been reported to present transient changes during cancer therapy, providing insight into the relationship between STMs and tumor progression [17]. Additionally, lung immune prognostic index (LIPI) has been proven to be a useful tool to help select advanced NSCLC patients who can benefit from immune checkpoint inhibitor (ICI) treatment [18]. Moreover, previous studies have indicated that the lymphocyte-monocyte ratio (LMR), neutrophil-lymphocyte ratio (NLR), and platelet-lymphocyte ratio (PLR) have vital prognostic value in various kinds of solid tumors, such as gastric cancer and endometrial cancer [19, 20]. Systemic immune-inflammation index (SII) was also shown to be a predominant prognostic factor in patients with NSCLC, [21] gastroesophageal adenocarcinoma, [22] hepatocellular carcinoma, [23] and pancreatic cancer [24]. Our study attempted to select the most effective predictors from all of the above variables to establish a scoring system to predict PFS for EGFR-positive NSCLC patients.

## Materials and methods

### Patients

This retrospective clinical study included 208 consecutive patients with advanced EGFR-positive NSCLC treated with icotinib between Januaray 2017 and October 2020 at the Wuhan Union Hospital. Patients were excluded if they did not have laboratory examination results within 30 days prior to the onset of icotinib therapy, or if the follow-up data were missing. Patients received icotinib monotherapy or in combination with other adjuvant treatments, such as chemotherapy and radiotherapy. Patient demographics, tumor characteristics, and laboratory biomarkers including age, sex, Eastern Cooperative Oncology Group performance status (ECOG PS), smoking status, tumor histology, EGFR mutation type, tumor stage, metastases, adjuvant treatment, several laboratory combined indices, and three STMs were collected from patients’ medical records. Uncommon EGFR mutations were those other than the exon 19 deletion (19Del), exon 21 L858R (L858R), and compound mutations. The combined indices were calculated as follows: LMR, lymphocyte/monocyte; NLR, neutrophil/lymphocyte; PLR, platelet/lymphocyte; SII, platelet*neutrophil/lymphocyte; prognostic nutritional index (PNI), albumin (g/L) + 5×lymphocyte (10^9^/L); albumin-globulin ratio (AGR), albumin/globulin. LIPI was determined based on the derived neutrophils/(leukocytes minus neutrophils) ratio (dNLR) and level of lactate dehydrogenase (LDH) [18]. The three STMs were CEA, CA-125 and CA19-9.

This study was conducted in accordance with the International Council for Harmonization Guidelines for Good Clinical Practice and the Declaration of Helsinki. The Ethics Committee of Wuhan Union Hospital approved the study protocol and waived the need for informed consent due to the retrospective study design (No. S363).

### Assessment of outcomes

The primary endpoint was survival information with PFS and the secondary endpoint was response rate. The best overall response (complete response (CR), partial response (PR), stable disease (SD), progressive disease (PD), or not evaluated), objective response rate (ORR$=$CR$+$PR), and disease control rate (DCR$=$CR$+$PR$+$SD) were evaluated according to the revised Response Evaluation Criteria in Solid Tumors, version 1.1 (RECIST ver.1.1) guidelines. PFS was defined as the period from the start of icotinib treatment until disease progression or death. The last follow-up was on August 21, 2021.

### Statistical analysis

For baseline characteristics, continuous variables were expressed as a mean ± standard deviation (SD) or median (interquartile range (IQR)), whereas categorical variables were expressed using relative frequencies and proportions. The optimal cut-off values of continuous variables for one-year PFS were identified with the maximal Youden index according to receiver operating characteristic (ROC) curve analysis. The selection of the final prognostic predictors was performed in two steps: [1] Twenty variables, including age, sex, ECOG PS, smoking status, histology, EGFR mutation, disease stage, brain metastases, bone metastases, pleural metastases, LIPI, LMR, NLR, PLR, SII, PNI, AGR, CEA, CA-125, and CA19-9, were enrolled in the least absolute shrinkage and selection operator (LASSO) regression analysis. [2] Thereafter, a Cox proportional hazard model was constructed using the features selected in the LASSO regression model to estimate the hazard ratio (HR) and 95% confidence interval (CI). The final scoring system was validated using a five-fold cross-validation. Survival curves for PFS were plotted using the Kaplan–Meier method. Comparisons of variables between the two groups were performed. As appropriate, the Student’s t-test or the Mann–Whitney U-test was performed for continuous variables, and the chi-square test or Fisher’s exact test for categorical variables. A *P-*value $<$0.05 was statistically significant.

## Results

### Baseline characteristics

**Fig. 1:**
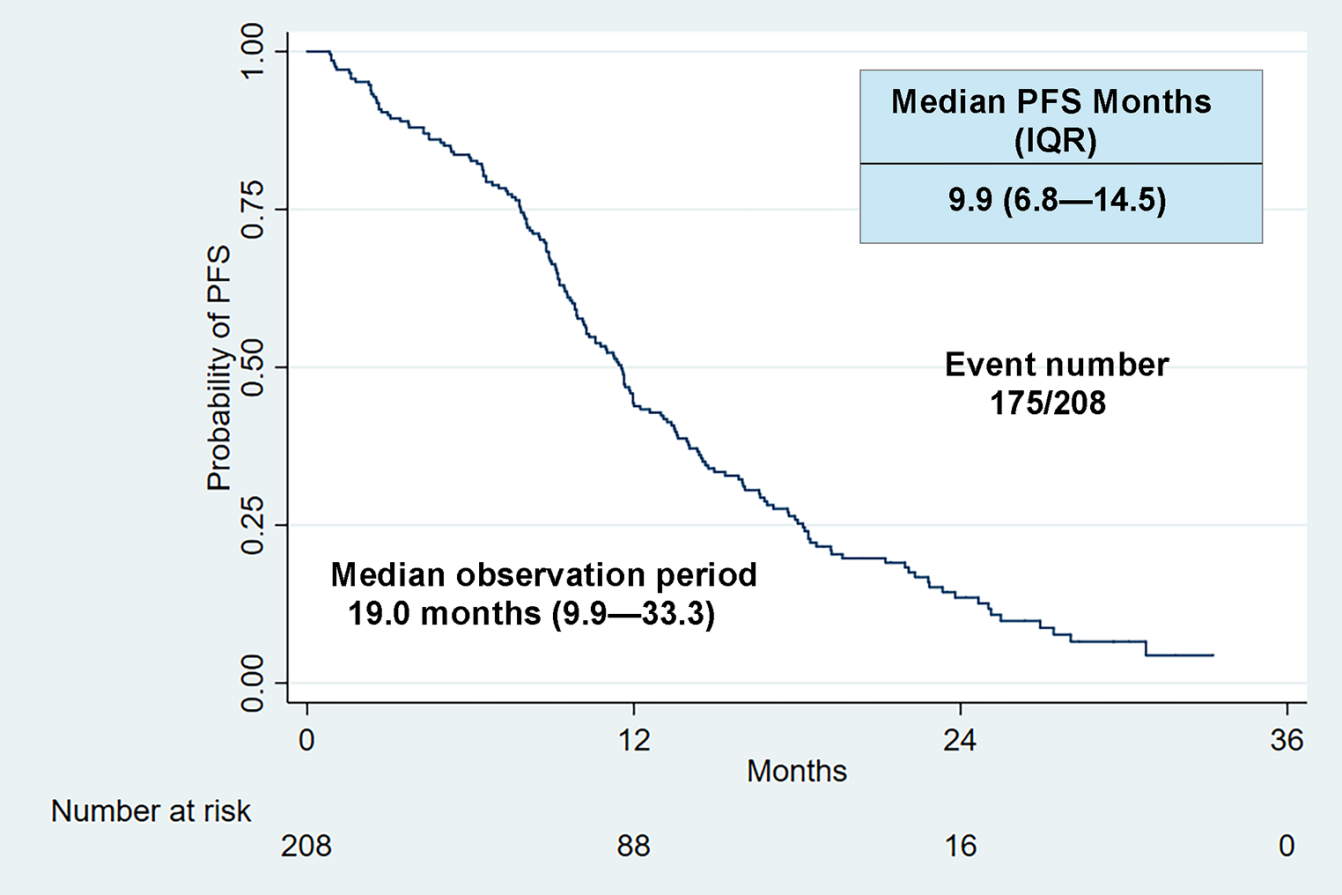
Kaplan-Meier curves of progression-free survival (PFS). IQR: interquartile range

Among the 208 patients, the majority were male (60.1%), non-smoker (74.0%), adenocarcinoma (97.1%), and received adjuvant treatment (60.1%) while taking icotinib (Table 1). Only 9 (4.3%) patients had ECOG PS of 2 or higher, and 18 (8.6%) expressed uncommon EGFR mutations. The mean age was 58.4 years (SD ± 10.5). 51.0% of patients presented with bone metastases, while 31.7% and 29.8% presented with brain and pleural metastases, respectively. PFS events occurred in 175 patients with a median follow-up duration of 19.0 months (range: 9.9–33.3 months) and a median PFS of 9.9 months (IQR: 6.8–14.5). The one-year PFS rate was 55.8% among all patients (Fig. 1). The ORR was 36.1%, and the DCR was 67.3% (Table S1).

**Table 1:** Patients’ baseline characteristics

Characteristics	Results	%
Age, years, Mean ± SD	58.4 ± 10.5	
Sex		
Male	125	60.1
Female	83	39.9
ECOG PS		
0–1	199	95.7
2–3	9	4.3
Smoking status		
Never	154	74.0
Current or former	54	26.0
Histology		
Adenocarcinoma	202	97.1
Other carcinomas	6	2.9
EGFR mutation status		
Exon 19 deletion	100	48.1
Exon 21 L858R	90	43.3
Uncommon mutation	18	8.6
Disease stage		
III	17	8.2
IV	191	91.8
Tumour metastases		
Brain	66	31.7
Bone	106	51.0
Pleural	62	29.8
Other	40	19.2
Adjuvant treatment		
Yes	107	60.1
No	71	39.9
LIPI		
0	120	58.3
1	66	32.0
2	20	9.7
LMR, Median (IQR)	3.18 (2.29–4.46)	
NLR, Median (IQR)	3.04 (2.04–4.95)	
PLR, Median (IQR)	157.18 (119.28-224.37)	
SII, Median (IQR)	675.68 (403.14-1133.79)	
PNI, Mean ± SD	45.89 ± 5.67	
AGR, Mean ± SD	1.52 ± 0.30	
CEA, µg/L, Median (IQR)	16.77 (4.75–94.30)	
CA-125, U/ml, Median (IQR)	38.80 (18.50-104.80)	
CA19-9, U/ml, Median (IQR)	8.85 (4.10–38.40)	

SD: standard deviation; IQR: interquartile range; ECOG PS: Eastern Cooperative Oncology Group performance status; EGFR: epidermal growth factor receptor; LIPI: lung immune prognostic index; LMR: lymphocyte-monocyte ratio; NLR: neutrophil-lymphocyte ratio; PLR: platelet-lymphocyte ratio; SII: systemic immune-inflammation index; PNI: prognostic nutritional index; AGR: albumin-globulin ratio; CEA: carcinoembryonic antigen; CA: carbohydrate antigen.

### LASSO regression analysis

Firstly, the optimal cutoff values of age, LMR, NLR, PLR, SII, PNI, A/G, CEA, CA-125 and CA19-9 for one-year PFS were determined by ROC curve assessment using the Youden index (Table 2). Each continuous variable was converted into two groups based on the optimal cut-off value. Twenty associated characteristic variables were included in the LASSO regression analysis. Seven potential factors, including age, bone metastases, LMR, SII, PNI, CEA, and CA19-9 with nonzero regression coefficients after the shrinkage process, were selected to be most strongly associated with the one-year PFS (Table 3). The LASSO coefficient paths of one-year PFS for all the initial twenty variables and the optimal lambda (λ) are shown in Figure S1.

**Table 2:** Cutoff values of continuous variables

Variables	Cutoff value	Sensitivity (%)	Specificity (%)	Youden index
Age, years	57	55.17	58.62	0.138
LMR	1.10	7.76	98.85	0.066
NLR	7.59	12.07	94.25	0.063
PLR	192.68	36.21	73.56	0.098
SII	1873.21	15.52	95.40	0.109
PNI	45.50	50.00	64.37	0.144
AGR	1.30	33.62	74.71	0.083
CEA, µg/L	31.12	51.40	72.50	0.239
CA-125, U/ml	92.90	34.95	77.33	0.123
CA19-9, U/ml	18.40	47.00	74.67	0.217

LMR: lymphocyte-monocyte ratio; NLR: neutrophil-lymphocyte ratio; PLR: platelet-lymphocyte ratio; SII: systemic immune-inflammation index; PNI: prognostic nutritional index; AGR: albumin-globulin ratio; CEA: carcinoembryonic antigen; CA: carbohydrate antigen.

**Table 3:** Risk factors of one-year progression-free survival selected by LASSO regression model

Starting variables	Selected variables	Regress. Coeff.
Age	X	-0.034
Sex		
ECOG PS		
Smoking status		
Histology		
EGFR mutation status		
Disease stage		
Brain metastases		
Bone metastases	X	0.050
Pleural metastases		
LIPI		
LMR	X	-0.032
NLR		
PLR		
SII	X	0.019
PNI	X	-0.003
AGR		
CEA	X	0.058
CA-125		
CA19-9	X	0.052

LASSO: least absolute shrinkage and selection operator; ECOG PS: Eastern Cooperative Oncology Group performance status; EGFR: epidermal growth factor receptor; LIPI: lung immune prognostic index; LMR: lymphocyte-monocyte ratio; NLR: neutrophil-lymphocyte ratio; PLR: platelet-lymphocyte ratio; SII: systemic immune-inflammation index; PNI: prognostic nutritional index; AGR: albumin-globulin ratio; CEA: carcinoembryonic antigen; CA: carbohydrate antigen.

### Selection of the final three prognostic predictors to form the ABC-Scoring system

**Fig. 2:**
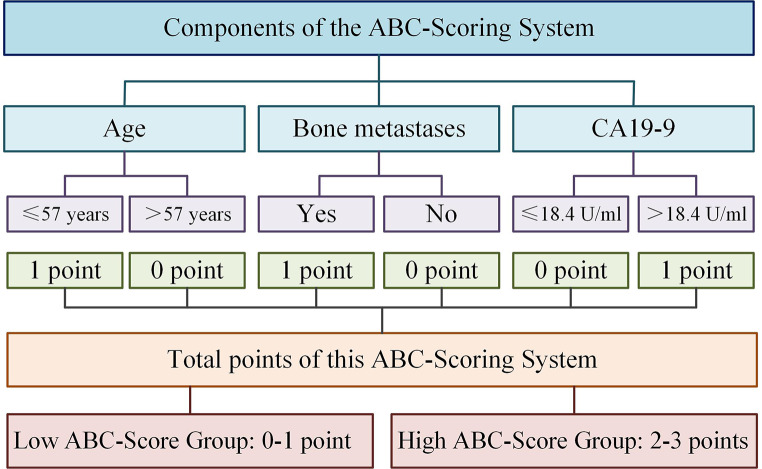
The detailed definition and grouping items of ABC-Score.

COX regression analysis was performed using the above seven selected variables, and among them, age, bone metastases, and CA19-9 showed significant statistical differences (Table 4). Therefore, the three predictors constituted the ABC-Scoring system to predict the one-year PFS for advanced EGFR-positive NSCLC patients treated with icotinib as EGFR-TKI targeted therapy. Age ≤ 57 were scored as 1, otherwise sored as 0; Having bone metastases were scored as 1, otherwise sored as 0; CA19-9 > 18.4U/ml were scored as 1, otherwise sored as 0. Patients were divided into two groups: the low ABC-Score group (score 0–1) and the high ABC-Score group (score 2–3) (Fig. 2). The one-year PFS rates of patients in the low ABC-Score group and the high ABC-Score group were 55.7% (95%CI: 46.6–64.0%) and 25.7% (95%CI: 16.8–35.6%), respectively. Additionally, the comparison of baseline characteristics between the low and high ABC-Score groups is shown in Table S2. In addition to the three predictors, only disease stage and other tumor metastases showed significant differences between the two ABC-Score groups (*P* < 0.05). The ORR and DCR of the low and high ABC-Score groups were 37.50% and 35.53%, and 72.12% and 61.85%, respectively.

**Table 4:** Estimated hazard ratio of risk factors for one-year progression-free survival

Variables		HR	95% CI	*P* value
Age	≤ 57	1.00	-	-
	> 57	0.62	(0.41–0.93)	0.021
Bone metastases	No	1.00	-	-
	Yes	1.86	(1.21–2.85)	0.005
LMR	≤ 1.10	1.00	-	-
	> 1.10	0.66	(0.30–1.48)	0.318
SII	≤ 1873.21	1.00	-	-
	> 1873.21	1.32	(0.73–2.40)	0.353
PNI	≤ 45.50	1.00	-	-
	> 45.50	0.84	(0.55–1.29)	0.431
CEA	≤ 31.12 µg/L	1.00	-	-
	> 31.12 µg/L	1.53	(0.99–2.35)	0.055
CA19-9	≤ 18.40 U/ml	1.00	-	-
	> 18.40 U/ml	1.68	(1.10–2.57)	0.016

HR: hazard ratio; CI: confidence interval; LMR: lymphocyte-monocyte ratio; SII: systemic immune-inflammation index; PNI: prognostic nutritional index; CEA: carcinoembryonic antigen; CA: carbohydrate antigen.

### Predictive performance of ABC-Score

**Fig. 3:**
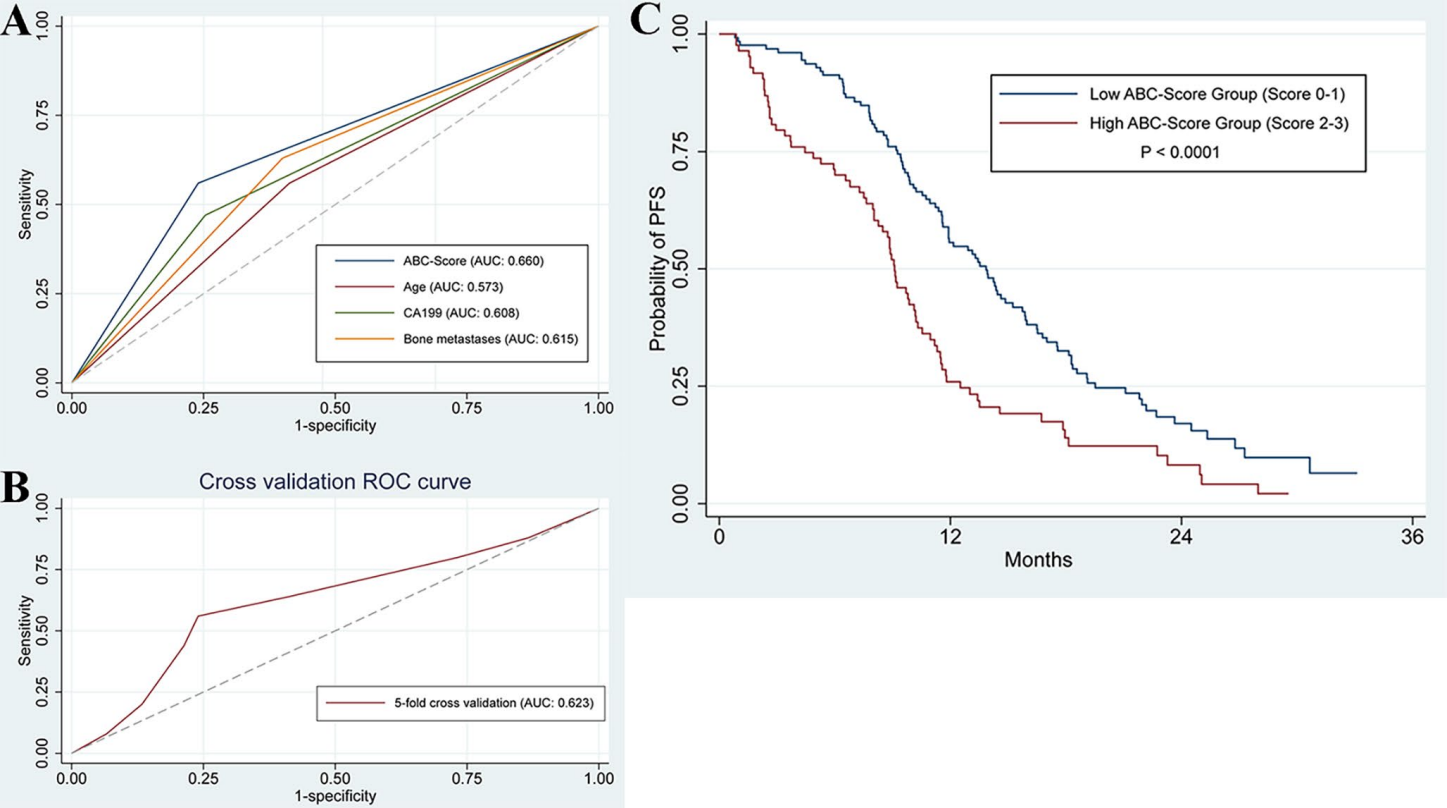
Predictive performance of ABC-Score. (A) ROC curves predicting one-year PFS of the ABC-Score, age, CA19-9 and bone metastases. (B) ROC curves of the 5-fold cross validation for the ABC-Score. (C) Kaplan–Meier curves for PFS between high ABC-Score group and low ABC-Score group

**Fig. 4:**
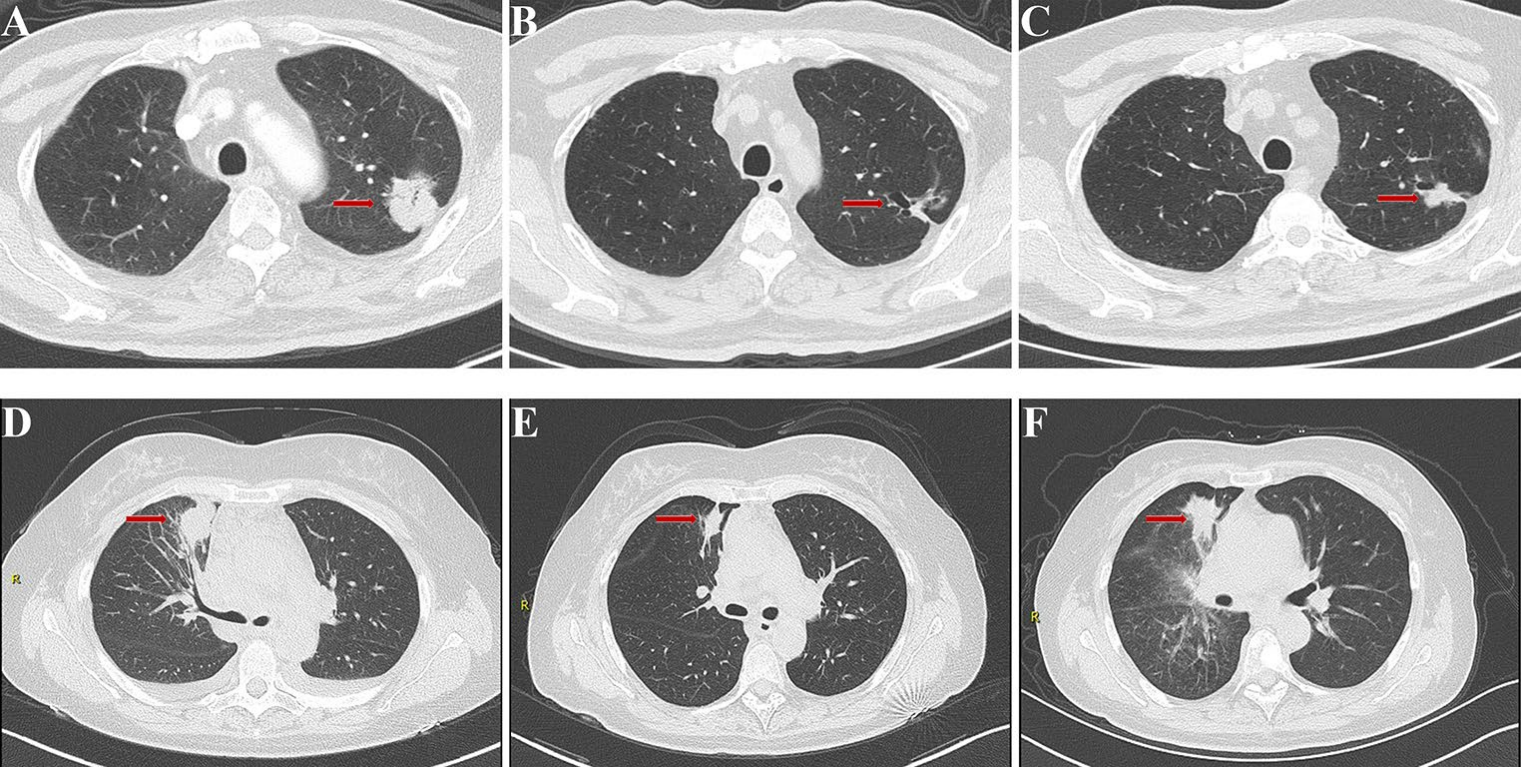
Example of CT images from pre-treatment to progression of two patients with different ABC-Scores. CT images before icotinib treatment (A), at the time of partial response (B), and at the time of disease progression (C) of a 60-year-old woman with the ABC-Score equal to 1. CT images before icotinib treatment (D), at the time of partial response (E), and at the time of disease progression (F) of a 57-year-old woman with the ABC-Score equal to 3

ROC analysis was used to access the predictive performance of the ABC-Score for one-year PFS rate of advanced NSCLC patients treated with icotinib. Results of the analysis showed the following: age (area under the curve (AUC) = 0.573), bone metastases (AUC = 0.615), and CA19-9 (AUC = 0.608). Compared with the three predictors individually, the combined ABC-Score (AUC = 0.660) showed a better predictive accuracy (Fig. 3A). The ABC-Scoring system performed well in the five-fold cross-validation (AUC = 0.623) (Fig. 3B). Kaplan–Meier survival analysis indicated that advanced NSCLC patients in the low ABC-Score group showed better PFS (*P* < 0.0001) than those in the high ABC-Score group (Fig. 3C). Representative CT images before icotinib treatment, at the time of partial response, and at the time of disease progression in two patients with different ABC-Scores are shown in Fig. 4. The PFS of a 60-year-old woman with an ABC-Score equal to 1 was 17 months, while the PFS of a 57-year-old woman with an ABC-Score of 3 was 8 months.

### Subgroup analysis based on adjuvant treatment and EGFR mutation types

Subgroup analysis was performed based on adjuvant treatment and the presence of two common EGFR mutations. It showed that the ABC-Score revealed similar superior predictive performance for one-year PFS for the subgroup with adjuvant treatment (AUC = 0.629) and the subgroup without adjuvant treatment subgroup (AUC = 0.678). There was no significant difference of PFS shown in Kaplan–Meier survival analysis between patients with and without adjuvant treatment (*P* = 0.9908) (Figure S2). In addition, subgroup analysis of the two types of common EGFR mutations indicated that predictive performance of the ABC-Score was superior for both, the EGFR 19Del subgroup (AUC = 0.679) and the EGFR L858R subgroup (AUC = 0.636). There was no significant difference of PFS noted in the Kaplan–Meier survival analysis between these two subgroups (*P* = 0.2580) (Figure S3).

## Discussion

The median PFS of all the enrolled patients treated with icotinib in our study was 9.9 months, which is similar to previous studies [13, 25]. PD events occurred in 175 NSCLC patients, with 116 events occurring within one year. The primary aim of this study was to select several key predictors and construct a scoring system to determine whether advanced EGFR-positive NSCLC patients have a greater probability for PFS beyond one year with icotinib as EGFR-TKI targeted therapy. Potential variables included patient demographics, tumor characteristics, nutritional and systemic inflammatory combined indices, and serum tumor markers. The final ABC-Score consisted of three predictors: age, bone metastases and CA19-9. In addition, the ROC curves indicated that the scoring system had a better predictive performance than the three predictors alone.

It is universally acknowledged that age is a key risk factor not only for cancer, but many other diseases. While elderly people are commonly considered to have poor healthy conditions, the age ≤ 57 years was a risk factor in our study. It has been reported that younger patients with lung cancer tend to have a worse OS than older group [26]. Moreover, young NSCLC patients are more likely to have advanced stage of disease at diagnosis than older patients [27]. Metastasis is one of the most important features and a major cause of cancer deaths in advanced NSCLC with the advent of diverse extrapulmonary metastatic lesions, among which the most frequent sites are brain, bone and liver [28]. Approximately 40–50% of lung cancer patients have brain metastases, and about 30% of patients simultaneously develop metastasis to the bone when diagnosed with brain metastases from the lung [28, 29]. Patients with lung cancer with liver and bone metastases have been shown to have worse survival than those with other sites of metastasis [28]. In the mean time, previous studies have found that a younger age is an independent risk factor for brain and lymph node metastases in patients with NSCLC [30, 31]. Our study has similar results, with age ≤ 57 years and bone metastasis decreasing the probability for one-year PFS in advanced *EGFR*-positive NSCLC patients.

One of the enabling characteristics of cancer that has gained authoritative certification is tumor-promoting inflammation, which makes a significant contribution to the activation of core programs in the microenvironment [32]. There is growing evidence that inflammation plays a crucial role in all stages of tumorigenesis and progression. In fact, an increasing number of inflammatory indices and biomarkers have been used to predict the efficacy of immunotherapy and have acted as prognostic factors for cancer patients. Thompson et al. created a weighted score combining epithelial-to-mesenchymal transition (EMT) and inflammatory signatures, which showed high accuracy in predicting responses to ICI therapy in advanced NSCLC patients [33]. Initially, PNI was defined to assess the baseline nutritional status to predict the risk of postoperative complications for malnourished patients with gastrointestinal cancers [34]. Subsequently, PNI level was demonstrated to be associated with prognosis of diverse tumors, tumor stage, and tumor-infiltrating lymphocytes status [35, 36]. Similarly, AGR was shown to be related to OS and lymph node metastasis for cancer patients [37]. In addition, a previous study demonstrated that worsening nutritional status, which was derived from the measures of PNI and body mass index (BMI), indicated poor immunotherapy outcomes for advanced cancer patients [38]. However, none of the combined nutritional and systemic inflammatory indexes enrolled in our study stood out from the statistic analysis.

Although STMs are characterized by low specificity, precise measurement of a panel of STMs can considerably improve the value of early diagnosis and efficacy monitoring of cancers [39]. Another issue is that an increasing of STMs during the disease is closely related to tumour progression. However, changes in STMs within the first four weeks of TKI therapy for advanced NSCLC patients may be unreliable according to Noonan et al [40]. Chen et al. found that preoperative serum CA19-9 could predict the recurrence free survival of patients with lung squamous cell carcinoma [41]. Nevertheless, the pre-treatment level of CA19-9 combined with the other two predictors showed great efficacy to determine the predictive performance of icotinib in this research. More research is needed to confirm the exact changes in STMs that can be considered as signs of tumor progression.

It has been reported that icotinib can easily pass through the cell membrane and blood-brain barrier because of its high permeability to tissue [42]. Liu et al. suggested that pemetrexed combined with icotinib in different sequences had different anticancer capabilities in NSCLC cells, and that treatment with pemetrexed followed by icotinib was the best sequence [43]. Another study demonstrated that icotinib combined with antiangiogenic drugs inhibited tumor growth significantly without increasing the toxicity compared to monotherapy [44]. Additionally, the antiangiogenesis effect was elevated by this combination. Combined therapeutic strategies usually have stronger antitumor effects, owing to the potential effects and interactions between various antitumor drugs. However, the subgroup analysis between the subgroups with and without adjuvant treatment showed no significant differences on PFS. One of the potential reasons for this was the heterogeneity of the detailed patterns of combination treatment with icotinib among patients and the limited size of the cohort in our study. Further exploration about the mechanisms of drug combinations is essential in terms of the complex biological factors and signaling pathways in tumor formation.

The following limitations of this study should be noted. First, it was a single-center retrospective study, which means that the data are less representative. There was no external validation cohort that could be used to further verify the performance of the ABC-Score. Second, information about OS was unavailable due to the long observation period. Therefore, PFS was chosen as the primary endpoint. Third, the heterogeneity of treatment, mainly caused by the concrete chemotherapeutic or radiotherapeutic adjuvant treatment regimens, was not avoided. The comparison of adjuvant treatment between the low and high ABC-Score groups was not statistically significant (*P* = 0.100). Fourth, this study did not analyze other inflammation-associated markers (such as various immune cells and cytokines) to determine the relationship between inflammation and icotinib efficacy. We plan to improve our research in the future. On the one hand, a multi-center, prospective study is necessary to testify the accuracy of the ABC-Score to predict the efficacy of icotinib among all-stage EGFR-positive NSCLC patients. On the other hand, whether the ABC-Score can be used for predicting the efficacy of other EGFR-TKI-targeted therapies, such as osimertinib, is worthy of future exploration.

To sum up, this study demonstrated that age, bone metastases and CA19-9 can be used to construct an ABC-Score to predict the efficacy of icotinib as an EGFR-TKI targeted therapy for advanced EGFR-positive NSCLC patients. Patients in the low ABC-Score group had a higher probability for PFS beyond one year. Scoring patient before icotinib treatment may influence future therapeutic strategies and guide the efficacy monitoring examinations. Simultaneously, doctors can adopt more individualized strategies according to the precise evaluation of each patient. To validate our results, a prospective study design and external validation cohort are warranted in our future research.

## Electronic supplementary material

Below is the link to the electronic supplementary material.

Supplementary Material 1

## Data Availability

The data that support the findings of this study are available from the corresponding author upon reasonable request.
