# Supplementary material for: Prognosis prediction of icotinib as targeted therapy for advanced EGFR-positive non–small cell lung cancer patients
Source: Invest New Drugs. 2023 May 4;41(3):463–72. doi: 10.1007/s10637-023-01329-8 (PMC10289924; doi:10.1007/s10637-023-01329-8)
Supplement: Supplementary file 1 — Supplementary Material 1 [file 10637_2023_1329_MOESM1_ESM.docx]

**Supplementary Tables**

**Table S1.** Overall response of all enrolled patients

| Overall response | Overall population, N=208 (%) |
| --- | --- |
| CR | 1 (0.5%) |
| PR | 74 (35.6%) |
| SD | 65 (31.2%) |
| PD | 43 (20.7%) |
| NE | 25 (12.0%) |
| ORR | 36.1% |
| DCR | 67.3% |

N: number; CR: complete response; PR: partial response; SD: stable disease; PD: progressive disease; NE: not evaluated; ORR: objective response rate; DCR: disease control rate.

**Table S2.** Patients charateristics between the two-category ABC-Score groups

| Characteristics | Score 0-1 (n=125) | Score 2-3 (n=83) | *P* value |
| --- | --- | --- | --- |
| Age, years, Mean ± SD | 61.0 ± 9.9 | 54.33 ± 10.16 | <0.001 |
| Sex | | | 0.746 |
| Male | 51 (40.80%) | 32 (38.55%) |  |
| Female | 74 (59.20%) | 51 (61.45%) |  |
| ECOG PS | | | 0.160 |
| 0-1 | 120 (96.00%) | 79 (95.18) |  |
| 2-3 | 5 (4.00%) | 4 (4.82) |  |
| Smoking status | | | 0.411 |
| Never | 90 (72.00%) | 64 (77.11%) |  |
| Current or former | 35 (28.00%) | 19 (22.89%) |  |
| Histology | | | 1.000 |
| Adenocarcinoma | 121 (96.80%) | 81 (97.59%) |  |
| Other carcinomas | 4 (3.20%) | 2 (2.41%) |  |
| EGFR mutation status | | | 0.240 |
| Exon 19 deletion | 55 (44.00%) | 45 (54.22%) |  |
| Exon 21 L858R | 60 (48.00%) | 30 (36.14%) |  |
| Uncommon mutation | 10 (8.00%) | 8 (9.64%) |  |
| Disease stage | | | 0.013 |
| III | 15 (12.00%) | 2 (2.41%) |  |
| Ⅳ | 110 (88.00%) | 81 (97.59%) |  |
| Tumour metastases | | | |
| Brain | 35 (28.00%) | 31 (37.35) | 0.156 |
| Bone | 32 (25.60%) | 74 (89.16) | <0.001 |
| Pleural | 38 (30.40%) | 24 (28.92%) | 0.819 |
| Other | 18 (14.40%) | 22 (26.51%) | 0.030 |
| Adjuvant treatment | | | 0.100 |
| Yes | 56 (54.90%) | 51 (67.11%) |  |
| No | 46 (45.10) | 25 (32.89) |  |
| LIPI | | | 0.108 |
| 0 | 79 (63.71%) | 41 (50.00%) |  |
| 1 | 33 (26.61%) | 33 (40.24%) |  |
| 2 | 12 (9.68%) | 8 (9.76%) |  |
| Overall response |  |  | 0.059 |
| CR | 0 (0%) | 0 (0%) |  |
| PR | 39 (37.50%) | 27 (35.53%) |  |
| SD | 36 (34.62%) | 20 (26.32%) |  |
| PD | 15 (14.42%) | 23 (30.26%) |  |
| NE | 14 (13.46%) | 6 (7.89%) |  |
| ORR | 39 (37.50%) | 27 (35.53%) |  |
| DCR | 75 (72.12%) | 47 (61.85%) |  |
| LMR, Median (IQR) | 3.37 (2.48-4.94) | 3.00 (2.03-4.25) | 0.062 |
| NLR, Median (IQR) | 3.11 (2.07-4.97) | 2.93 (2.02-4.82) | 0.609 |
| PLR, Median (IQR) | 157.04 (125.37-220.29) | 157.32 (115.38-232.43) | 0.845 |
| SII, Median (IQR) | 676.87 (430.00-1162.53) | 662.32 (374.29-1083.87) | 0.647 |
| PNI, Mean ± SD | 45.99 ± 5.54 | 45.75 ± 5.89 | 0.759 |
| A/G, Mean ± SD | 1.53 ± 0.30 | 1.50 ± 0.29 | 0.620 |
| CEA, μg/L, Median (IQR) | 15.84 (4.30-92.64) | 24.04 (6.69-95.91) | 0.289 |
| CA-125, U/ml, Median (IQR) | 38.20 (18.20-93.10) | 45.50 (23.00-114.90) | 0.295 |
| CA19-9, U/ml, Median (IQR) | 6.25 (3.45-17.10) | 25.00 (6.05-70.60) | <0.001 |

SD: standard deviation; IQR: interquartile range; ECOG PS: Eastern Cooperative Oncology Group performance status; EGFR: epidermal growth factor receptor; LIPI: lung immune prognostic index; CR: complete response; PR: partial response; SD: stable disease; PD: progressive disease; NE: not evaluated; ORR: objective response rate; DCR: disease control rate; LMR: lymphocyte-monocyte ratio; NLR: neutrophil-lymphocyte ratio; PLR: platelet-lymphocyte ratio; SII: systemic immune-inflammation index; PNI: prognostic nutritional index; AGR: albumin-globulin ratio; CEA: carcinoembryonic antigen; CA: carbohydrate antigen.

**Supplementary Figures**


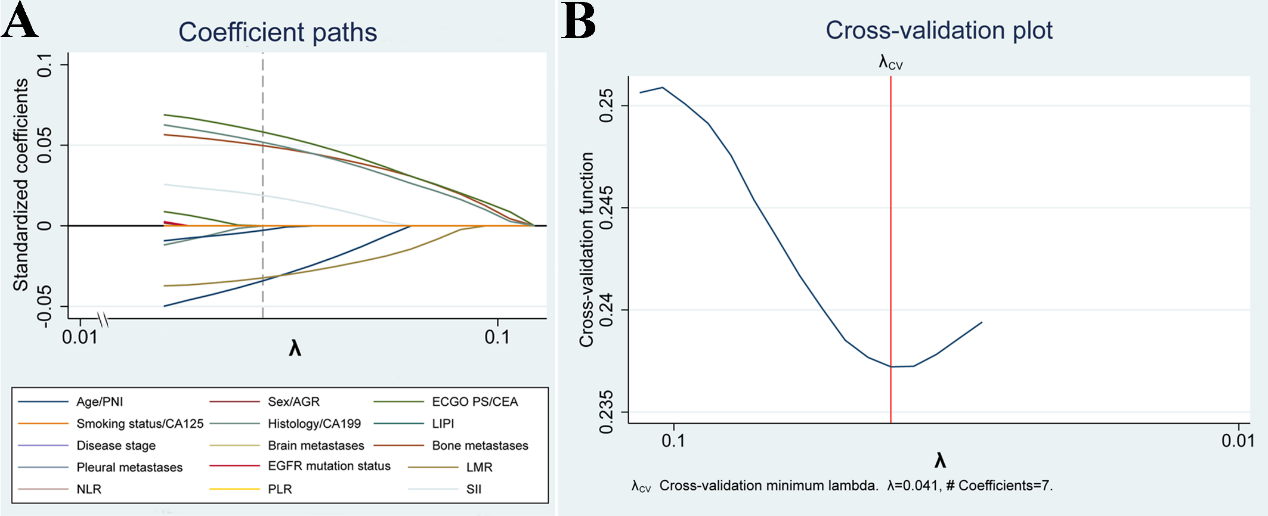


**Figure S1.** Factor selection using least absolute shrinkage and selection operator (LASSO) method. (A) LASSO coefficient profiles of selected features shown by lambda parameters. (B) The cross-validation plot with seven nonzero coefficients selected by optimal lambda.


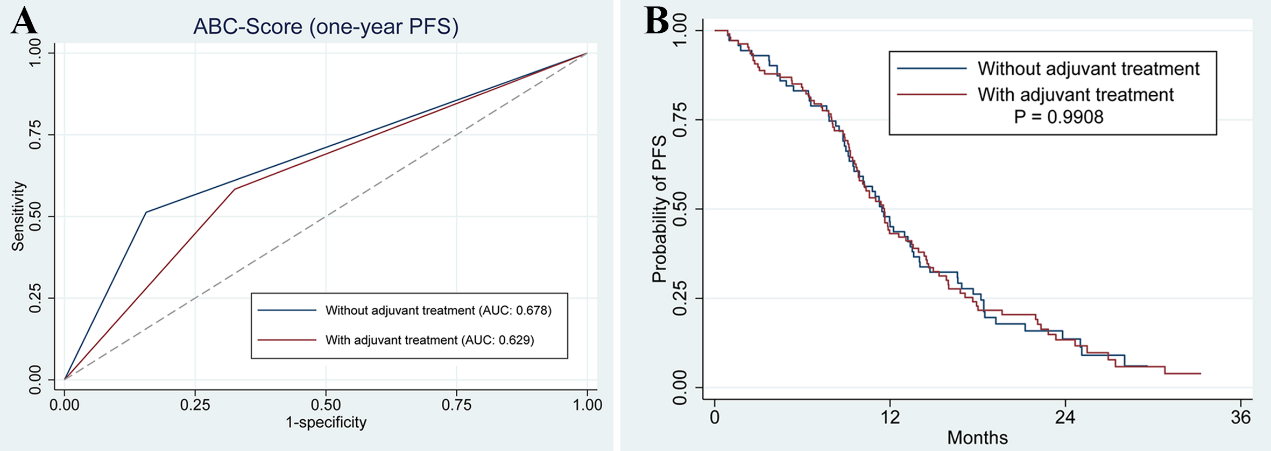


**Figure S2.** (A) ROC curves predicting one-year PFS of the ABC-Score for patients with and without adjuvant treatment. (B) Kaplan–Meier curves for PFS between patients with and without adjuvant treatment.


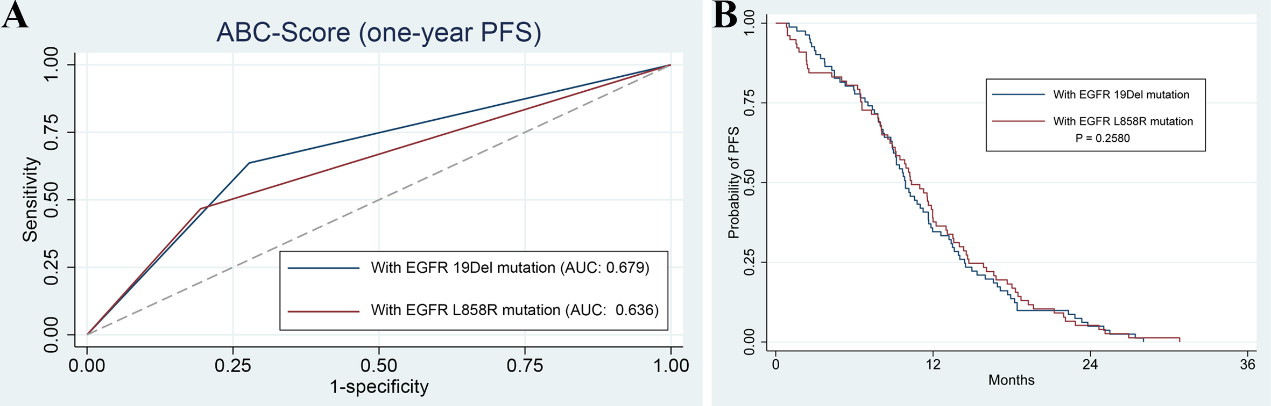


**Figure S3.** (A) ROC curves predicting one-year PFS of the ABC-Score for patients with EGFR 19Del mutation and EGFR L858R mutation. (B) Kaplan–Meier curves for PFS between patients with EGFR 19Del mutation and EGFR L858R mutation.
